# Unveiling Periapical Actinomycosis: A Rare Extraradicular Infection

**DOI:** 10.7759/cureus.79907

**Published:** 2025-03-02

**Authors:** Wei Chun Yeoh, Spoorthi Ravi Banavar, Jothi Raamahlingam Rajaran, Kai Ling Siew

**Affiliations:** 1 Sungai Pelek Dental Clinic, Ministry of Health Malaysia, Sungai Pelek, MYS; 2 Clinical Oral Health Sciences, School of Dentistry, IMU University, Kuala Lumpur, MYS; 3 Private Dental Practice, Perfect Healthcare Dental Clinic, Kuala Lumpur, MYS; 4 Private Dental Practice, SKL Endodontic Dental Clinic, Kuala Lumpur, MYS

**Keywords:** actinomyces spp, apicoectomy, extraradicular infection, oral microbiome, periapical actinomycosis

## Abstract

Actinomycosis is an insidious infection caused by the facultative anaerobic Gram-positive bacterium *Actinomyces*, commonly found in the oral microbiome. Among its manifestations, periapical actinomycosis stands out as a rare subtype within cervicofacial actinomycosis, speculated to play a role in the persistence of periapical radiolucencies following root canal therapy. Instances of this occurrence often arise from disruptions in epithelial continuity, stemming from surgical procedures, trauma, or prior infections, paving the way for deep microbial infiltration. A 35-year-old woman presented with persistent pain and swelling in the premolar region of her mandible. Examination unveiled a radiolucent lesion in an endodontically treated premolar. The tooth remained symptomatic, and the draining sinus tract persisted despite multiple attempts at disinfecting and re-medicating the canal. A subsequent apicoectomy was performed, yielding promising outcomes, with satisfactory periapical healing observed at both the 6- and 12-month follow-up evaluations.

## Introduction

Post-treatment apical periodontitis is a common sequela of endodontic therapy, often resulting from the failure to eradicate infection and achieve microbial control within the root canal system [1]. However, post-treatment apical periodontitis can also occur in teeth that have undergone seemingly meticulous endodontic procedures, suggesting that factors beyond intraradicular infection may contribute to disease persistence. Studies indicate that 5-15% of cases with pre-operative apical periodontitis exhibit persistent disease despite strict adherence to established endodontic protocols [2, 3].

The persistence of microbial infection is the primary etiology of post-treatment apical periodontitis, with bacteria either surviving within the root canal system (persistent or secondary intraradicular infection) or extending beyond the apical foramen into periradicular tissues (extraradicular infection). Extraradicular infections are particularly challenging, as they may involve microbial biofilms that resist host immune responses and antimicrobial agents. The pathogenesis of extraradicular infections is complex, with two potential mechanisms proposed [4]: 1) Dependent extraradicular infections occur when bacteria from an active intraradicular infection continuously infiltrate and colonize periradicular tissues. This interplay between the intraradicular and extraradicular components sustains infection, but conventional root canal therapy alone is often sufficient to resolve it; 2) Independent extraradicular infections are theorized to persist in the absence of an active intraradicular infection and may not respond to conventional root canal therapy, requiring additional surgical intervention.

Strong evidence supporting the existence of truly independent extraradicular infections remains limited. Ricucci et al. analyzed teeth diagnosed with post-treatment apical periodontitis and found no definitive cases of extraradicular infection occurring in isolation [5]. To date, only a few cases have proposed that extraradicular infections might develop independently of intraradicular bacteria and contribute to post-treatment apical periodontitis, although such occurrences remain rare and lack robust validation [6].

This report presents the case of a 35-year-old female with persistent post-treatment apical periodontitis, where an extraradicular infection played a critical role in disease progression, underscoring the diagnostic and therapeutic challenges associated with this condition.

## Case presentation

A 35-year-old Chinese female was referred for the management of a recurrent infection in her lower right second premolar. She reported intermittent swelling and pus drainage from the affected region. Her medical history showed no significant contributing factors.

**Figure 1:**
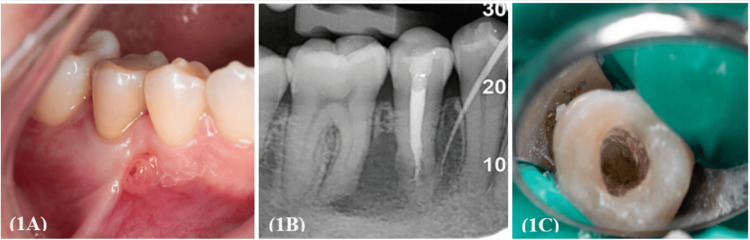
Pre-operative clinical and radiographic findings with access cavity preparation (1A) Presence of intraoral swelling and sinus tract in relation to tooth 45. (1B) Pre-operative intraoral periapical radiograph revealing a well-defined periapical radiolucency, with sinus tract tracing localized to tooth 45. (1C) Access cavity preparation performed under rubber dam isolation.

Upon clinical examination, swelling and a sinus tract were evident near tooth 45 (Figure 1A). Tooth 45 exhibited tenderness to both percussion and palpation, with periodontal probing depth within normal limits. Pulp sensibility tests for teeth 44 and 46 elicited positive responses, thereby excluding both teeth as the etiological sources of the periapical infection. An intraoral periapical radiograph displayed a clearly delineated periapical radiolucency extending from the periapical region of the right mandibular second premolar to the right mandibular first molar, with the sinus tract tracing localized to the root-treated tooth 45 (Figure 1B). The root canal filling appeared underfilled, being at least 3-4 mm short of the radiographic apex. Substandard obturation creates an environment conducive to persistent intraradicular infection, as inadequate disinfection, insufficient sealing, and residual necrotic tissue serve as microbial reservoirs, perpetuating periapical pathology. The tooth was therefore diagnosed as previously treated 45 associated with chronic apical abscess. Preliminary differential diagnoses include periapical granuloma, radicular cyst, and extraradicular infection. The patient provided informed consent for the necessary endodontic retreatment of tooth 45. Posicaine® 200 (articaine hydrochloride 4% with epinephrine/adrenaline 1:200,000 injection; Novocol Pharmaceutical of Canada, Inc, Cambridge, Canada) was administered, followed by access cavity preparation under rubber dam isolation and aided by a dental operating microscope (DOM) (Figure 1C). 

**Figure 2:**
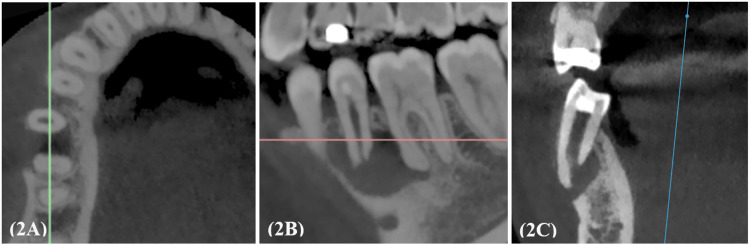
Cone-beam computed tomography (CBCT) 2A-2C: Periapical radiolucency associated with tooth 45 visualized in axial, sagittal, and coronal planes, respectively, demonstrating the extent of the lesion.

The previous root-filling material was removed using Gates Glidden burs and hand K-files (Dentsply Maillefer, Ballaigues, Switzerland), while residual gutta-percha (GP) and sealer remnants were dissolved using chloroform. Upon meticulous inspection under magnification and illumination, a large apical opening was evident. No crack lines or fractures were detected in the tooth structure. The working length was determined using the electronic apex locator Root ZX II (J. Morita, Kyoto, Japan). Subsequently, the root canal was thoroughly cleaned, shaped, and irrigated with copious amounts of 5.25% sodium hypochlorite (NaOCl) (Clorox Company, Broadway, Oakland, USA). The canal was then dressed with non-setting calcium hydroxide UltraCal™ XS (Ultradent Products, Inc, South Jordan, USA), and the tooth was temporized using Cavit® (3M ESPE, Seefeld, Germany) and GC Fuji 9 cement (GC, Tokyo, Japan). During the follow-up appointments, the tooth remained symptomatic upon percussion and palpation with non-resolving, persistent sinus tract over the buccal aspect of tooth 45, despite multiple re-medication attempts. Given the persistence of symptoms and the non-resolving infection, the possibility of an extraradicular infection was strongly considered. The patient was informed of the prognosis, and an apicoectomy was recommended for the eradication of lingering microbial infection. Cone-beam computed tomography (CBCT) revealed a well-defined osteolytic lesion extending from the periapical region of the right mandibular second premolar to the right mandibular first molar. The coronal plane of the CBCT scan revealed an open apex associated with tooth 45 (Figure 2A-2C).

**Figure 3:**
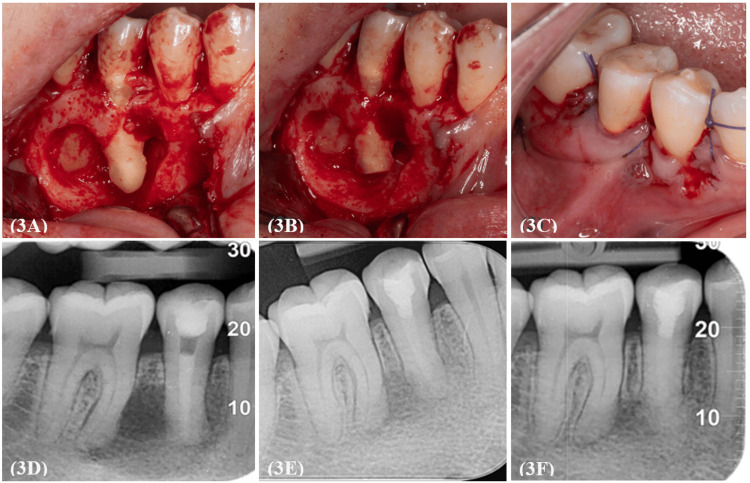
Peri-operative clinical photographs of periradicular surgery with post-operative and follow-up periapical radiographs (3A) Flap elevation revealed substantial resorption of the buccal cortical plate. (3B) Resection of 3 mm of the apical root to eliminate complex apical anatomical structures, including apical ramifications and lateral canals. (3C) Reapproximation of the flap with 4-0 Vicryl sutures. (3D) Immediate post-operative radiograph. (3E, 3F) Radiographic evidence of bone regeneration at the 6- and 12-month follow-up respectively, indicating periapical healing.

After performing another round of root canal disinfection, the canal was obturated with Biodentine™ (Septodont, Saint-Maur-des-Fossés, France), and the tooth was temporised. A full-thickness flap was raised from tooth 46 to 43, with a distal relieving incision. The resorption of the buccal cortical plate was evident upon flap elevation, eliminating the need for an osteotomy to create a surgical window (Figure 3A). Granulation tissue at the surgically exposed site was enucleated and curetted. Using a surgical carbide bur under copious saline irrigation, approximately 3 mm of the apical third of the root was resected to remove complex apical anatomical structures, such as apical ramifications and lateral canals, which are not amenable to thorough disinfection by conventional disinfection techniques (Figure 3B) [7]. Since the root filling material was clearly visible, clean, and homogeneously condensed, no retrograde root filling was placed. The wound was subsequently cleaned using 0.2% chlorhexidine. The flap was then repositioned, and sutures 4-0 Vicryl threads were used for closure (Figure 3C). Immediate post-operative periapical radiography was performed (Figure 3D). As part of post-surgical management, the patient was prescribed celecoxib** **200 mg OD, ibuprofen 400 mg TDS, paracetamol 1 g QID/PRN for 3 days and was instructed to rinse with a 0.2% chlorhexidine mouthwash twice daily for 1 week. After 7 days, the sutures were removed, and the patient's post-operative recovery was favourable with no complications. The patient returned for follow-up assessments after 6 and 12 months, revealing radiographic evidence of bone regeneration indicated by a reduction in periapical radiolucency and the absence of any clinical signs or symptoms (Figures 3E, 3F).

**Figure 4:**
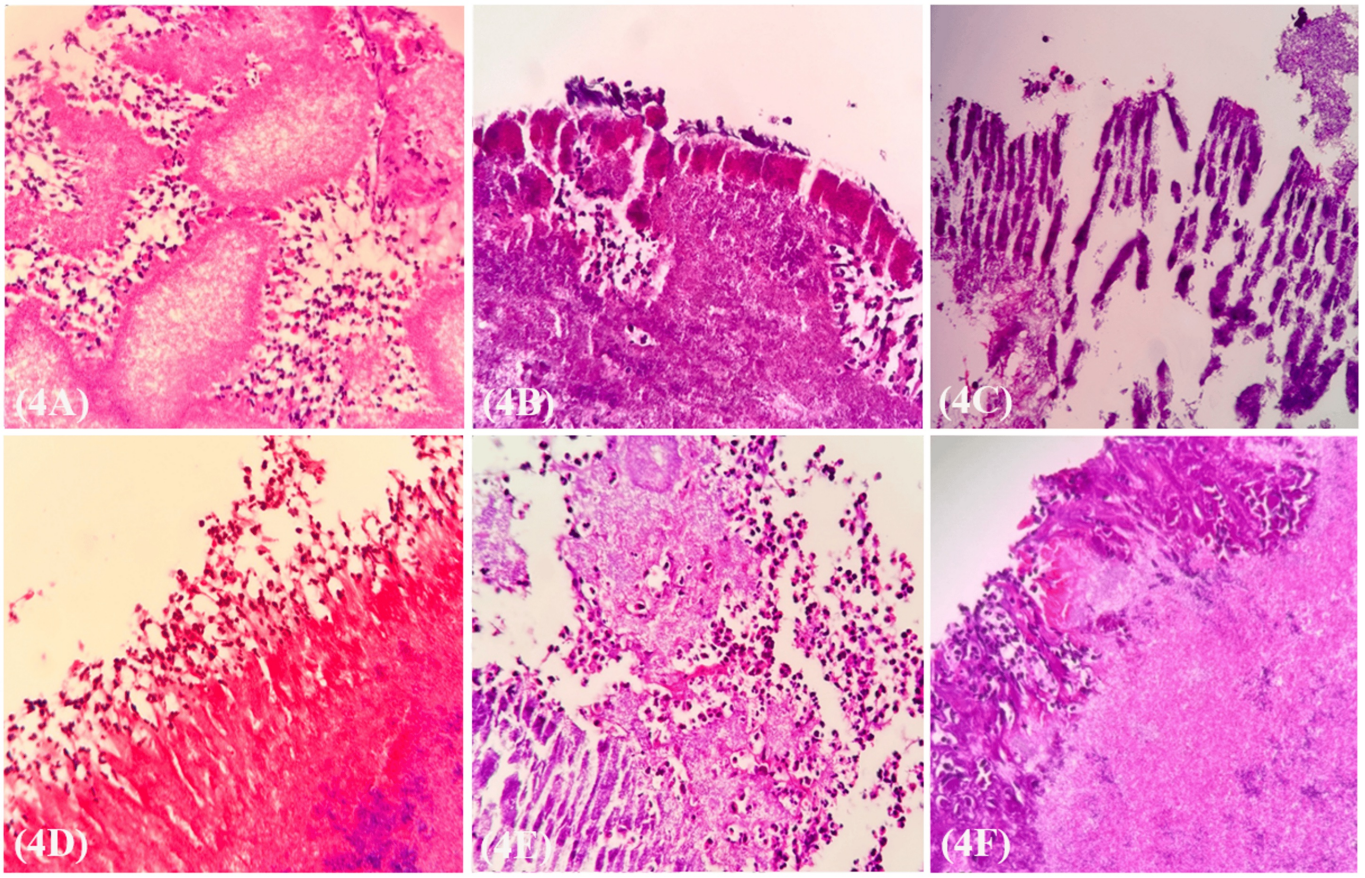
Histology panel Histology panel showing (4A) large bacterial colonies with peripheral radiating actinomycetes (H & E stain, 10x). (4B) High magnification shows the peripheral thick radiating clubs (H & E stain, 20x). (4C) Thick radiating clubs of actinomycetes organisms (H & E stain, 20x). (4D) Accumulation of leukocytes around bacterial colonies (H & E stain, 20x). (4E) Bacterial colonies mixed with leukocytes and thick radiating clubs (H & E stain, 20x). (4F) Dense bacterial colonies with extensive peripheral radiating clubs (H & E stain, 20x).

Histopathological analysis of the biopsied periapical lesion revealed hallmark features of actinomycotic infection. Hematoxylin and eosin (H&E) staining demonstrated large bacterial colonies with peripheral radiating filamentous structures, characteristic of *Actinomyces *spp (Figure 4A). At higher magnification, these colonies exhibited thick, radiating eosinophilic clubs, consistent with the Splendore-Hoeppli phenomenon - an intense eosinophilic rim encasing *Actinomyces *colonies, resulting from immune complex deposition and cellular debris accumulation (Figure 4B). Similar structures are observed in Figure 4C, reinforcing the presence of filamentous bacterial aggregates with peripheral radiating formations. A pronounced inflammatory response was evident. Figure 4D demonstrates dense infiltration of polymorphonuclear leukocytes encircling the bacterial colonies, indicative of an active immune reaction. Additionally, Figure 4E highlights bacterial aggregates interspersed with inflammatory cells, predominantly neutrophils, suggestive of a chronic suppurative response. The presence of densely packed bacterial colonies with extensive peripheral radiating clubs, characteristic of sulfur granules, is evident in Figure 4F.

## Discussion

Actinomycosis is a chronic granulomatous infection affecting both humans and animals, primarily caused by bacterial species belonging to the genera *Actinomyces *and *Propionibacterium *[8]. Among the recognized clinical forms - cervicofacial, thoracic, and abdominopelvic - cervicofacial actinomycosis is the most frequently encountered [9]. This predominance is largely attributed to the well-established presence of *Actinomyces *as part of the normal oral microbiota, particularly in individuals with compromised dentition. These bacteria exhibit a high affinity for dental plaques, periodontal pockets, gingival crevices, and tonsillar crypts, where they thrive as opportunistic colonizers [10]. *Actinomyces *species are frequently detected in carious dentin and are considered early colonizers of exposed pulp [11]. Notably, their presence has been strongly associated with cases of unhealed periapical lesions, a condition termed periapical actinomycosis [12].

The prevalence of periapical actinomycosis is believed to be underestimated, with literature suggesting it accounts for less than 5% of all periapical lesions [13]. However, this figure may not reflect the true incidence, as periapical surgical specimens are rarely submitted for histopathological examination when lesions are presumed to be typical periapical infections [14]. The majority of reported cases exist as isolated case reports, and large-scale epidemiological data remain limited [13]. The precise mechanism by which *Actinomyces *infiltrates the periapical region is not well understood. One prevailing hypothesis suggests that bacterial displacement from the oral cavity into the periapical tissues can occur during root canal treatment, particularly when strict aseptic protocols are not maintained. This theory is supported by the frequent association of periapical actinomycosis with endodontically treated teeth. Although periapical actinomycosis is commonly linked to teeth with a history of endodontic treatment, it can also develop in untreated teeth. Nair and Schroeder found *Actinomyces *in two of 45 periapical lesions without prior endodontic treatment [15]. The translocation of bacteria through a compromised periodontium has also been proposed as a contributing factor in the pathogenesis of periapical actinomycosis [16].

Among the *Actinomyces *species implicated in periapical actinomycosis, *Actinomyces israelii* and *Propionibacterium propionicum* are the most frequently isolated from periapical tissues of teeth that have failed to respond to conventional non-surgical endodontic therapy [17]. *Actinomyces *possess unique structural adaptations that contribute to their pathogenic potential, including a hydrophobic cell surface, a Gram-positive cell wall enveloped by a fuzzy outer layer, and fimbriae-like projections that facilitate bacterial aggregation [18]. These features promote the formation of cohesive bacterial colonies, which may provide protection against host immune responses. Meanwhile, although *P. propionicum* is recognized as a pathogen in actinomycotic infections, its precise mechanisms of virulence remain poorly understood.

Radiographically, periapical actinomycosis typically manifests as a well-defined radiolucent lesion in the apical region of a maxillary or mandibular tooth with a history of endodontic treatment. However, this radiographic appearance lacks specificity and may be mistaken for other odontogenic pathologies, such as periapical granulomas or abscesses. The presence of sulfur granules - compact bacterial colonies embedded in purulent exudate - serves as a key diagnostic feature in the histological examination. However, sulfur granules are not pathognomonic for actinomycosis, as they are only detected in 35-55% of cases and may also be observed in infections caused by *Nocardia*, *Streptomyces*, and *Peptostreptococcus *[19]. Tissue culture remains the most accurate method for diagnosing actinomycosis. However, its practical application is challenging due to the anaerobic nature of *Actinomyces *species, which makes culturing difficult [16]. In recent years, molecular and genetic techniques such as immunofluorescence, fluorescent in situ hybridization, and 16S rRNA analysis via polymerase chain reaction (PCR) have been increasingly employed to enhance diagnostic accuracy [9].

Traditionally, periapical actinomycosis has been considered a relatively mild manifestation of cervicofacial actinomycosis. Risk factors contributing to the dissemination of actinomycotic infection include mucosal disruption, compromised local or systemic immunity, suboptimal oral hygiene, facial trauma, previous head and neck radiation, and a history of oral surgical interventions [16]. Unlike the treatment protocol for cervicofacial disease, which typically involves surgical drainage and prolonged antibiotic therapy [20], the management of periapical actinomycosis follows a different approach. In most cases, resolution is achieved through endodontic treatment or retreatment aimed at eradicating the intraradicular infection [16]. When an extraradicular infection is suspected, periradicular surgery may be warranted to ensure complete debridement of infected tissues. In some cases, tooth extraction may be necessary to eliminate the persistent bacterial reservoir. While treatment outcomes for periapical actinomycosis are generally favorable, clinicians should remain vigilant, as untreated cases may progress to cervicofacial actinomycosis, necessitating a more aggressive therapeutic strategy.

## Conclusions

Periapical actinomycosis is a rare but significant cause of persistent post-treatment apical periodontitis, often resistant to conventional endodontic therapy. Its underreported prevalence highlights the need for heightened clinical awareness, particularly in cases of non-healing periapical lesions. A definitive diagnosis of periapical actinomycosis requires both histopathological analysis and microbiological confirmation, as its radiographic presentation mimics other periapical pathologies. Successful management typically requires a combination of surgical intervention and thorough debridement, underscoring the importance of recognizing extraradicular infections as potential contributors to endodontic failure.

## References

[1] Sjögren UL, Hägglund B, Sundqvist G, Wing K (1990). Factors affecting the long-term results of endodontic treatment. J Endod.

[2] Siqueira JF Jr, Rôças IN, Riche FN, Provenzano JC (2008). Clinical outcome of the endodontic treatment of teeth with apical periodontitis using an antimicrobial protocol. Oral Surg Oral Med Oral Pathol Oral Radiol Endod.

[3] Ricucci D, Russo J, Rutberg M, Burleson JA, Spångberg LS (2011). A prospective cohort study of endodontic treatments of 1,369 root canals: results after 5 years. Oral Surg Oral Med Oral Pathol Oral Radiol Endod.

[4] Siqueira Jr JF (2003). Periapical actinomycosis and infection with Propionibacterium propionicum. Endod Topics.

[5] Ricucci D, Siqueira JF Jr, Bate AL, Pitt Ford TR (2009). Histologic investigation of root canal-treated teeth with apical periodontitis: a retrospective study from twenty-four patients. J Endod.

[6] Ricucci D, Siqueira JF Jr, Lopes WS, Vieira AR, Rôças IN (2015). Extraradicular infection as the cause of persistent symptoms: a case series. J Endod.

[7] Kim S, Kratchman S (2006). Modern endodontic surgery concepts and practice: a review. J Endod.

[8] Könönen E, Wade WG (2015). Actinomyces and related organisms in human infections. Clin Microbiol Rev.

[9] Wong VK, Turmezei TD, Weston VC (2011). Actinomycosis. BMJ.

[10] Smego RA Jr, Foglia G (1998). Actinomycosis. Clin Infect Dis.

[11] Hoshino E, Ando N, Sato M, Kota K (1992). Bacterial invasion of non-exposed dental pulp. Int Endod J.

[12] Nair PN (2006). On the causes of persistent apical periodontitis: a review. Int Endod J.

[13] Hirshberg A, Tsesis I, Metzger Z, Kaplan I (2003). Periapical actinomycosis: a clinicopathologic study. Oral Surg Oral Med Oral Pathol Oral Radiol Endod.

[14] Kaplan I, Anavi K, Anavi Y, Calderon S, Schwartz-Arad D, Teicher S, Hirshberg A (2009). The clinical spectrum of Actinomyces-associated lesions of the oral mucosa and jawbones: correlations with histomorphometric analysis. Oral Surg Oral Med Oral Pathol Oral Radiol Endod.

[15] Nair PNR, Schroeder HE (1984). Periapical actinomycosis. J Endod.

[16] Dastgir R, Sohrabi M (2022). Periapical actinomycosis: a rare subdivision of cervicofacial actinomycosis, review of the literature, and a case report. Case Rep Dent.

[17] Happonen RP (1986). Periapical actinomycosis: a follow-up study of 16 surgically treated cases. Endod Dent Traumatol.

[18] Figdor D, Davies J (1997). Cell surface structures of Actinomyces israelii. Aust Dent J.

[19] Brook I (2008). Actinomycosis: diagnosis and management. South Med J.

[20] Moghimi M, Salentijn E, Debets-Ossenkop Y, Karagozoglu KH, Forouzanfar T (2013). Treatment of cervicofacial actinomycosis: a report of 19 cases and review of literature. Med Oral Patol Oral Cir Bucal.

